# Changing Patterns of Disease Severity in *Blastomyces dermatitidis* Infection, Quebec, Canada

**DOI:** 10.3201/eid2711.210552

**Published:** 2021-11

**Authors:** Alex Carignan, Chiheb Boudhrioua, Sandrine Moreira, Andrée Ann Pelletier, Kevin Dufour, Jacques Pépin, Catherine Allard, Dominique Marcoux, Philippe J. Dufresne

**Affiliations:** Université de Sherbrooke, Sherbrooke, Quebec, Canada (A. Carignan, A.A. Pelletier, K. Dufour, J. Pépin, C. Allard, D. Marcoux);; Laboratoire de Santé Publique du Québec, Sainte-Anne-de-Bellevue, Québec, Canada (C. Boudhrioua, S. Moreira, P.J. Dufresne)

**Keywords:** blastomycosis, *Blastomyces dermatitidis*, mortality, severity, epidemiology, fungi, Quebec, Canada

## Abstract

This retrospective multicenter cohort study assessed temporal changes in the severity and mortality rate of blastomycosis in Quebec, Canada, and identified risk factors for death in patients with blastomycosis in 1988–2016. The primary outcome was 90-day all-cause deaths. Among 185 patients, 122 (66%) needed hospitalization and 30 (16%) died. We noted increases in the proportion of severe cases, in age at diagnosis and in the proportion of diabetic and immunocompromised patients over time. Independent risk factors for death were age (adjusted odds ratio [aOR] 1.04, 95% CI 1.00–1.07), immunosuppression (aOR 4.2, 95% CI 1.5–11.6), and involvement of >2 lung lobes (aOR 5.3, 95% CI 1.9–14.3). There was no association between the *Blastomyces* genotype group and all-cause mortality. The proportion of severe cases of blastomycosis has increased in Quebec over the past 30 years, partially explained by the higher number of immunosuppressed patients.

## Abstract 2

The increase in severe cases might be related to an increased number of immunosuppressed patients; climate change might also play a role.

*Blastomyces dermatitidis*, a dimorphic fungus, causes a localized or disseminated pyogranulomatous fungal infection called blastomycosis. Various descriptive studies have shown predominantly pulmonary, skin, bone, and genitourinary involvement (*1*). The clinical spectrum of blastomycosis is wide, ranging from subclinical infection to critical cases of acute respiratory distress syndrome. Retrospective studies conducted in recent years have shown an increased incidence of blastomycosis in both Canada and the United States (*2*–*5*). Blastomycosis is endemic to the St. Lawrence River Valley; a study that focused on the incidence of blastomycosis in the province of Quebec, Canada (*6*), showed an increasing incidence from 1988 to 2011. Similar trends have been observed in several regions in the United States (*7*), although the underlying causes are poorly understood. The absence of robust reporting and lack of reportability in many jurisdictions may hamper the ability to know whether cases are increasing more broadly. Some authors suggested a possible relationship with certain climatic factors (*3*,*8*). In the wake of the increased incidence of blastomycosis in Quebec, clinicians have observed a possible worsening of the disease severity in patients, with occasional deaths.

This study aimed to assess temporal changes in the severity and mortality of blastomycosis in Quebec and to identify risk factors for blastomycosis-related deaths. In Quebec, all suspected *B. dermatitidis* isolates are sent for molecular species confirmation at the Quebec Public Health Laboratory (Laboratoire de Santé Publique du Québec; LSPQ; Sainte-Anne-de-Bellevue, QC, Canada), a Containment Level 3 laboratory. In addition, these strains are maintained in a strain biorepository. Therefore, we also aimed to establish a genetic diversity profile of the circulating *Blastomyces* strains in Quebec and assess whether major genotypes may be associated with increased disease severity or death. The institutional review board of Centre Intégré Universitaire de Santé et de Services Sociaux de l’Estrie—Centre Hospitalier Universitaire de Sherbrooke (CIUSSSE-CHUS) approved this study (project no. MP-31-2017-1597) and waived the need for individual informed consent because the study involved minimal to no risk to participants and this retrospective research could not practically be carried out without the waiver.

## Methods

We conducted a retrospective cohort study in 39 acute-care facilities, including community hospitals and academic centers, in Quebec. The study population included all patients with culture-confirmed *B. dermatitidis* infection who were treated as inpatients or outpatients. Data on *B. dermatitidis* cultures were extracted from the database (1988–2017) of the LSPQ, where all *B. dermatitidis* isolates are confirmed by an in-house detection PCR coupled with confirmatory sequencing of the internal transcribed spacer region and stored in the repository.

### Data Collection and Outcomes

Research assistants reviewed hospital records using a standardized questionnaire. The administrative region of residence was determined using postal codes (*9*). The medical history was delineated to calculate the Charlson Comorbidity Index (*10*), along with demographic, microbiological, clinical, and therapeutic data (antifungal drug, dose, route of administration, and start and end dates of treatment). Because there is no consensual definition of disseminated disease, we calculated the total number of organs involved, including the lungs. Immunosuppression included HIV infection, corticosteroid use, immunosuppressive therapy for inflammatory disease, chemotherapy, and transplantation. Severe cases were defined as patients with septic shock or acute respiratory distress syndrome or requiring mechanical ventilation, or any combination of those. The primary outcome was 90-day all-cause mortality.

### Genotyping

A total of 157 *Blastomyces* isolates, out of 185 in the LSPQ collection for which a medical record was available, were successfully grown on inhibitory mold agar or potato dextrose agar. We obtained whole-genome sequences (WGS) of 108 of those isolates using the Illumina MiSeq system (Illumina, https://www.illumina.com). In brief, we obtained DNA extracts from mycelium culture with the DNeasy PowerSoil extraction kit (QIAGEN, https://www.qiagen.com) and used for whole-genome pair-end sequencing with Nextera XT DNA Library Prep kit and reagent V3 (2 × 300 bp) kit (Illumina). We used the WGS data to detect single-nucleotide polymorphism (SNP) and genotypes calling by an in-house pipeline. Only high-quality SNPs having genotype calls (read depth >10 and mapping score >30) across all samples, from which >5% carried the minor allele, were stored. These SNPs were used to conduct population structure analysis with fastSTRUCTURE (https://rajanil.github.io/fastStructure) to identify major genetic groups present in the population (Appendix Figure).

### Statistical Analysis

We double-entered data into an electronic input tool, Research Electronic Data Capture (REDcap; Vanderbilt University, Nashville, TN, USA), and analyzed data using Stata version 15.1 for Mac (StataCorp, https://www.stata.com). Proportions were compared using the χ^2^ or Fisher exact test, as appropriate. We compared continuous variables by Wilcoxon rank-sum test. We excluded cases with missing data from the analyses. To establish risk factors for death, we selected the variables to be included in the multivariable unconditional logistic regression model by applying a 10% significance level after univariate analysis. We added variables one at a time and retained them only if they were found to be significant in the multivariable model based on the likelihood ratio test (p<0.05); the final model retained variables that significantly enhanced the fit of the model. To avoid potential confounding by indication, we excluded antifungal therapies from the multivariable analysis. We also excluded the variable representing case severity because it is on the causal pathway between the infection and the primary study outcome of 90-day all-cause mortality.

## Results

In total, 224 *B. dermatitidis–*positive culture results were extracted from the public health laboratory database. We included 185 cases of blastomycosis in 181 different patients with complete medical records in the analysis dataset of this study; of those, 157 isolates could be grown in culture, and 143 yielded high-quality WGS data. The remaining isolates were nonviable.

### Case Characteristics and Clinical and Radiologic Manifestations

Most patients were male (143/185; 77%), and the median age was 55 (interquartile range [IQR] 44–67) years. The median duration between the onset of the first symptoms and diagnosis was 56 (IQR 25–123) days. Pulmonary infection (n = 149; 81%) was predominant in this cohort, followed by cutaneous (n = 75; 40.5%), osteoarticular (n = 27, 15%), central nervous system (n = 8, 4%), and urinary (n = 11, 6%) involvement. Two or more organs were involved in 35% (64/185) of the patients. The most frequent symptoms when patients initially sought care were cough (n = 120; 65%) and dyspnea (n = 97; 52%). Fever was documented in 65 (35%) patients and weight loss in 62 (34%) patients. In patients with pulmonary infections, chest radiograph or computed tomography (CT) scan showed the involvement of >2 lobes in 64 (43%) patients. Radiologic manifestations on chest radiograph, chest CT, or both included, among others, nodular infiltrates (53; 36%), focal masses (27; 18%), and miliary patterns (n = 9; 6%). Furthermore, 122 patients (66%) needed hospitalization for a median duration of 15 (IQR 7–29) days. Of these, 37/122 (30%) patients were admitted to the intensive care unit (median duration 4 days, IQR 2–9 days). A severe form of blastomycosis was observed in 30/185 patients (16%), and the 90-day all-cause mortality was 16% (30/185).

### Temporal Changes in Characteristics, Treatment, and Outcomes of Blastomycosis

To further investigate temporal changes in blastomycosis, we compared the characteristics of case-patients from 1988–1997 (n = 33) with those identified in 1998–2007 (n = 57) and 2008–2017 (n = 95) (Table 1). We observed a significant increase over time in the age of patients with blastomycosis over the study periods (p = 0.02). In addition, we saw increases in the proportion of diabetic patients, immunocompromised patients, and patients with a Charlson Comorbidity Index score >3. Among the case-patients diagnosed during the 2008–2017 period, we noted an increasing use of the lipid formulations of amphotericin B over that of amphotericin B deoxycholate. There was no significant change in the distribution of *B. dermatitidis* genotypes over time: we observed an increase in the proportion of severe cases over the study periods (1988–1997, 1/33 [3%]; 1998–2007, 8/57 [15%]; 2008–2016, 21/95 [22%]; p = 0.03) (Figure), as well as in the 90-day all-cause mortality (1988–1997, 2/33 [6%]; 1998–2007, 11/54 [18%]; 2008–2016, 18/89 [19%]), but the differences between the study periods were not significant (p = 0.15).

**Table 1:** Characteristics of patients in study of blastomycosis in Quebec, Canada*

Characteristic	1988–1997, n = 33	1998–2007, n = 57	2008–2017, n = 95	p value
Median age, y (IQR)	47.4 (42.9–61.2)	50.0 (40.4–62.8)	58.9 (48.6–70.6)	0.02†
Sex				
F	11 (33)	13 (23)	18 (19)	
M	22 (67)	44 (77)	77 (81)	0.2
No. involved organs				
1	17 (52)	34 (60)	70 (74)	
>2	16 (48)	23 (40)	25 (26)	0.04
Underlying conditions				
Chronic obstructive pulmonary disease	6 (18)	7 (12)	21 (22)	0.3
Diabetes	1 (3)	9 (16)	22 (23)	0.03
Immunosuppression	2 (6)	11 (19)	31 (33)	0.005
Charlson Comorbidity Index				
0	20 (61)	28 (49)	33 (35)	
1–2	11 (33)	12 (21)	30 (32)	
>3	2 (6)	17 (30)	32 (34)	0.01
First antifungal administered				
Amphotericin B, lipid formulations	0	5 (9)	20 (21)	
Amphotericin B, deoxycholate	5 (15)	6 (11)	6 (6)	
Azole	20 (61)	33 (58)	58 (61)	
No treatment	8 (24)	13 (23)	11 (12)	0.008‡
*Blastomyces* genotype group				
I	1 (6)	1 (2)	1 (2)	
II	3 (17)	11 (24)	11 (24)	
III	4 (22)	13 (29)	14 (30)	
IV	10 (56)	20 (44)	20 (44)	0.9
Severe illness	1 (3)	8 (15)	21 (22)	0.03
90-day all-cause mortality	2 (6)	10 (18)	18 (19)	0.2

*Values are no. (%) except as indicated. IQR, interquartile range. †By Mood's equality of medians test. ‡By Fisher exact test.

**Figure:**
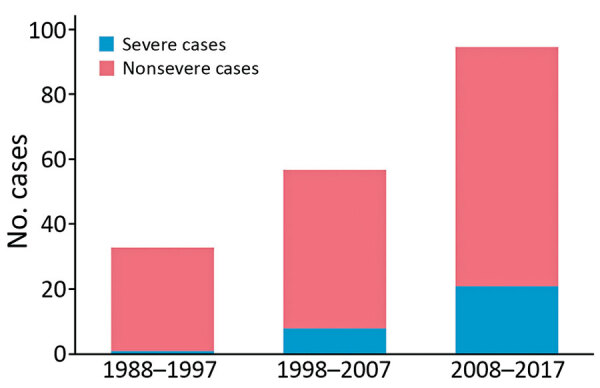
Cases of *Blastomyces dermatitidis* infection by severity, Quebec, Canada, 1988–2017.

### Risk Factors for Mortality

Several variables were associated with mortality in univariate analysis but were no longer significant after adjusting for confounders. There was no significant association between genotype and mortality. The independent risk factors associated with mortality included age (p = 0.04), immunosuppression (p = 0.005), and the involvement of >2 lung lobes on chest radiograph (p = 0.001) (Table 2).

**Table 2:** Risk factors for 90-day all-cause mortality among patients with blastomycosis, Quebec, Canada*

Risk factor	Survived, n = 155		Died, n = 30	Crude odds ratio (95% CI)		p value		Adjusted odds ratio (95% CI)		p value	
Study period											
1988–1997	31 (94)		2 (6)	1							
1998–2007	47 (82)		10 (18)	3.3 (0.7–16.1)		0.1		2.7 (0.5–15.3)		0.3	
2008–2017	77 (81)		18 (19)	3.6 (0.8–16.6)		0.1		1.2 (0.2–6.5)		0.8	
Median no. days of symptoms before diagnosis (IQR)	71 (26–141)		37 (18–45)	0.99 (0.99–1.00)		0.04		0.99 (0.99–1.00)		0.07	
Median age, y (IQR)	54 (44–64)		62 (53–71)	1.04 (1.01–1.07)		0.004		1.04 (1.00–1.07)		0.04	
Sex											
F	34 (81)		8 (19)								
M	121 (85)		22 (15)	0.8 (0.3–1.9)		0.6					
Charlson Comorbidity Index score											
0	76 (94)		5 (6)								
1–2	43 (81)		10 (19)	3.5 (1.1–16.1)		0.03					
≥3	36 (71)		15 (29)	6.3 (2.1–18.8)		0.001					
Underlying condition										
Diabetes	23 (72)		9 (28)	2.5 (1.00–6.04)		0.05					
COPD	27 (79)		7 (21)	1.4 (0.6–3.7)		0.5					
Immunosuppression	27 (61)		17(39)	6.2 (2.7–14.3)		<0.001		4.2 (1.5–11.6)		0.005	
No. organs involved											
1–2	105 (87)		16 (13)								
>2	50 (78)		14 (22)	1.8 (0.8–4.1)		0.1					
Radiologic manifestations											
No. lobes											
0–2	112 (93)		8 (7)								
>2	43 (66)		22 (34)	7.2 (3.0–17.3)		<0.001		5.3 (1.9–14.3)		0.001	
Miliary presentation	6 (67)		3 (33)	2.8 (0.7–11.7)		0.2					
First antifungal received											
Amphotericin, lipid formulations	20 (80)		5 (20)	1							
Amphotericin B, deoxycholate	13 (76)		4 (24)	1.2 (0.3–5.5)		0.08					
Azole	107 (96)		4 (4)	0.1 (0.04–0.61)		0.008					
No treatment	15 (47)		17 (53)	4.5 (1.4–15.1)		0.01					

*Values are no. (%) except as indicated. COPD, Chronic obstructive pulmonary disease; IQR, interquartile range.

### Genotype

A total of 108 *Blastomyces* isolates were available for WGS genotyping. Using WGS data, we detected a total of 97,403 high-quality SNPs among all the isolates sequenced. For the purpose of this study, we used SNP genotype calls across all isolates for structure analysis to underline the major genetic groups present in the population and to assess if they could correlate with disease outcome and severity. We identified 4 main genotypes in the population. Genotype IV (n = 50; 46%) was most frequently isolated, followed by genotype III (n = 31; 28%) and II (n = 25; 23%). Genotype I was isolated infrequently in this study (n = 3; 2%); it formed an outlier group representing the cryptic species *B. gilchristii*. We saw a correlation between administrative regions and genotypes (Table 3); however, we detected no association between genotypes and the proportion of severe cases or all-cause mortality. Sequences from this study have been deposited in the National Center for Biotechnology Information (project no. PRJNA752385).

**Table 3:** Characteristics of patients according to *Blastomyces* genotype groups, Quebec, Canada*

Characteristic	Genotype I, n = 3	Genotype II, n = 25	Genotype III, n = 31	Genotype IV, n = 49	p value
Quebec administrative region					
Bas-St-Laurent	0	0	0	0 (10)	
Saguenay-Lac-St-Jean	0	0	0	1 (2)	
Capitale-Nationale	0	0	0	13 (27)	
Mauricie	0	0	1 (3)	5 (10)	
Estrie	1 (33)	0	25 (81)	4 (8)	
Montréal	1 (33)	3 (12)	3 (10)	3 (6)	
Outaouais	1 (33)	10 (40)	0	0	
Chaudière-Appalaches	0	0	1 (3)	5 (10)	
Laval	0	1 (4)	0	0	
Lanaudière	0	7 (28)	0	0	
Laurentides	0	1 (4)	0	0	
Montérégie	0	3 (12)	1 (3)	4 (8)	
Centre-du-Québec	0	0	0	9 (18)	<0.001†
Severe case					
No	3 (100)	20 (80)	27 (87)	43 (86)	
Yes	0	5 (20)	4 (13)	7 (14)	0.8†
90-day all-cause mortality					
No	3 (100)	19 (76)	26 (84)	41 (82)	
Yes	0	6 (24)	5 (16)	9 (18)	0.9†

*Values are no. (%) except as indicated. †By Fisher exact test.

## Discussion

This study demonstrated a gradual modification in the clinical characteristics and underlying conditions associated with *B. dermatitidis* infection in Quebec, Canada, over the last 3 decades, 1988–2017. We documented an increase in the proportion of severe cases and an increase in the age and proportion of diabetic and immunocompromised patients. The 90-day all-cause mortality rate remained stable at ≈20% for the last 2 decades despite this change in disease severity. Because blastomycosis is often misdiagnosed as bacterial pneumonia initially (*11*), and given this changing pattern of disease severity, clinicians in blastomycosis-endemic regions must recognize the possibility of *Blastomyces* infection earlier to avoid delays in both diagnosis and treatment initiation.

The increased proportion of severe cases observed within our cohort may be related to changes in the underlying characteristics of the affected patients. We observed an increase in the proportion of immunocompromised patients. The prevalence of immunosuppressed adults in Quebec is unknown; an estimated 2.7% of US adults self-reported that they were immunosuppressed in 2013 (*12*), and this number is thought to be increasing because of both greater life expectancy among immunosuppressed adults and new indications for immunosuppressive therapies (*13*,*14*). Furthermore, population aging and increasing prevalence of chronic disease and multiple underlying conditions within the population of Quebec may explain the increased proportion of severe cases that were observed since 1998 (*15*). In addition, there was a similar increase in the prevalence of diabetes in Quebec, from 4.7% in 2000–2001 to 7.2% in 2014–2015 (*16*). Because our definition of severe cases included, among others, the use of mechanical ventilation, the increase in the proportion of severe cases may also be partially explained by changes leading to increasing use of mechanical ventilation over the years (*17*).

We observed an overall mortality rate of 16.2% in this study cohort, which is higher than the mortality rate observed in recently published large cohorts. A US nationwide study found an overall in-hospital mortality rate of 6.9% (*18*), and an overall case-fatality rate in a cohort of 671 cases in Minnesota during 1999–2018 was 10% (*19*). In a systematic review and meta-analysis, the mortality estimate for general clinical cases of blastomycosis was 6.6% overall (*20*); substantial heterogeneity between the included studies was mainly attributed to inconsistent definitions of mortality. We used 90-day all-cause mortality, a more robust outcome than other studies that used outcomes such as attributable mortality, which might explain the higher mortality rate in our findings compared with previously published results. Despite the increasing proportion of severe cases, we observed that the case-fatality ratio remained stable over the past 20 years. This observation may reflect improvements in severe sepsis management (*21*) or mechanical ventilation strategies (*22*), such as extracorporeal membrane oxygenation, for blastomycosis-related acute respiratory distress syndrome (*23*). Extracorporeal membrane oxygenation use increased substantially since 2002 (*24*); this treatment has been shown to decrease deaths in adults with severe acute respiratory failure (*25*). In addition, we documented the gradual replacement of amphotericin B deoxycholate by lipid formulations of amphotericin B. Despite its well-demonstrated effectiveness, amphotericin B deoxycholate is associated with renal toxicity (*26*). Patients with AIDS who had disseminated histoplasmosis and were treated with liposomal amphotericin B have demonstrated better clinical outcomes compared with patients who were treated with the deoxycholate forms (*27*). The increasing use of lipid formulations of amphotericin B may have contributed to the improved patient outcomes in this study, although a head-to-head comparison of amphotericin B formulations for *B. dermatitidis* infection has not been performed in clinical trials. Our study was not adequately powered to verify this hypothesis.

We used WGS to characterize the complete genome of our isolates. The high reproducibility and discriminatory power of WGS (*28*) enabled us to detect a strong association between genotype groups and the region where patients lived, where we assumed they acquired infection. These findings are similar to those of a study in Canada (*29*) that showed that the populations of both *Blastomyces* species were associated with major freshwater drainage. Other studies have used polymorphic microsatellite markers to genotype *B. dermatitidis* and have suggested potential associations between the clinical phenotype and genetic groups of *B. dermatitidis*. Frost et al. found that SNP alleles were substantially different in cases of pulmonary and disseminated disease (*30*) and identified associations between the group 1 genotype (*B. gilchristii)* and pulmonary infection and between the group 2 genotype and disseminated disease (*31*). We had a very limited number of *B. gilchristii* infections in our study population and were therefore unable to assess the potential of these 2 groups to cause disseminated diseases within the cohort. We identified 4 main distinct genetic groups that correlate with the geographic origins, but we did not find any association between the genotype and the proportion of severe cases or mortality rate. These results indicate that virulence of *B. dermatitidis* is not correlated with its population genetic structure in Quebec. Nevertheless, we cannot exclude the possibility that virulence may be controlled by specific regions or alleles in the genome. In this case, other approaches could be more suitable such as genome-wide association mapping which reveals SNPs strongly associated with a given virulence trait.

The main limitation of our study is its retrospective nature, which could introduce information bias. We were able to minimize this bias by including on the study team specialized research assistants and infectious diseases fellows who ensured the completeness of data collection. Moreover, this study had limited power to assess the potential factors that could affect mortality rate (e.g., increasing use of lipid formulations of amphotericin B). Furthermore, the cases we analyzed in this study were restricted to patients for whom a positive *Blastomyces* culture was sent to the provincial health laboratory. Given that the diagnosis of *Blastomyces* infection is not limited to culture results, this leads to an underestimation of cases and may have created bias in our data.

The proportion of severe cases of blastomycosis in Quebec has increased over the past 30 years. These changes could be explained in part by the higher proportion of immunosuppressed patients, as well as the older age of infected persons. The study data do not support an increasing virulence of *B. dermatitidis* strains in Quebec. Future studies may help understand whether climate changes or more specific genetic determinants may have played a role in the emergence of more severe *B. dermatitidis* infections, the incidence of which has tripled over time.

Appendix Additional information about changing patterns of disease severity in Blastomyces *dermatitidis* infection, Quebec, Canada.
