# Supplementary material for: Changing Patterns of Disease Severity in Blastomyces dermatitidis Infection, Quebec, Canada
Source: Emerg Infect Dis. 2021 Nov;27(11):2810–7. doi: 10.3201/eid2711.210552 (PMC8544988; doi:10.3201/eid2711.210552)
Supplement: Appendix — Additional information about changing patterns of disease severity in Blastomyces dermatitidis infection, Quebec, Canada. [file 21-0552-Techapp-s1.pdf]

Article DOI: https://doi.org/10.301/eid2711.210552

Changing Patterns of Disease Severity in *Blastomyces dermatitidis* Infection, Quebec, Canada

Appendix

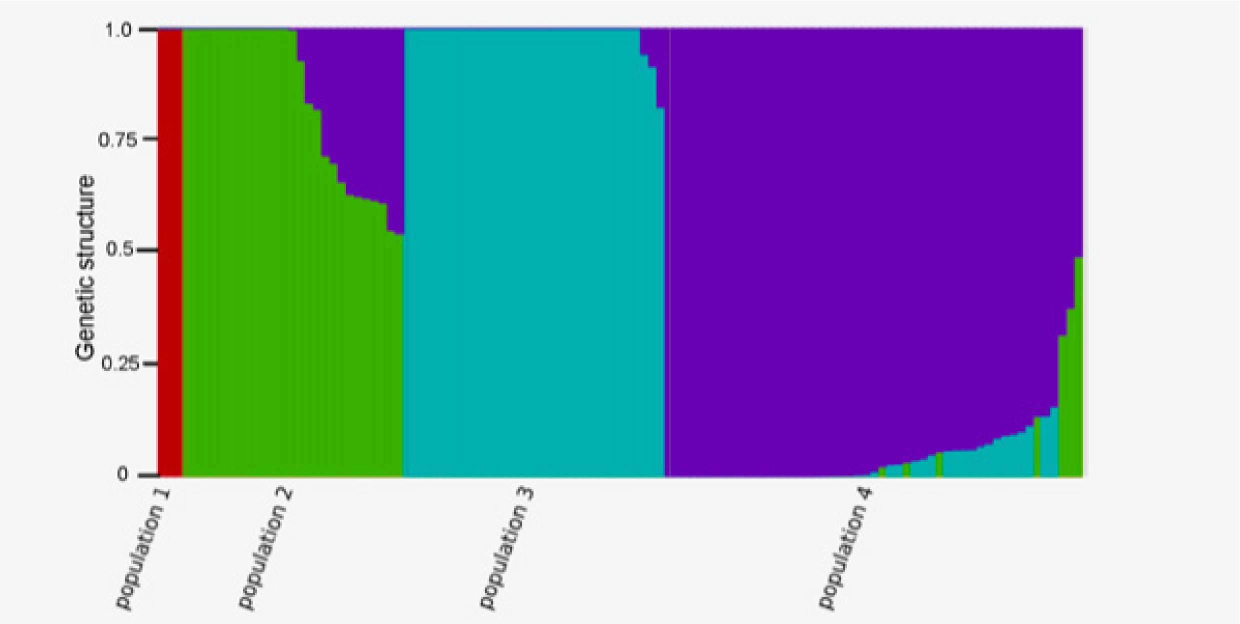

Appendix Figure. Population structure analysis of *Blastomyces dermatitidis* fungi based on 97,403 SNPs genotype calls across 108 strains. Each vertical line represents a strain and each color a separate population.
